# Correlation between centromere protein-F autoantibodies and cancer analyzed by enzyme-linked immunosorbent assay

**DOI:** 10.1186/1476-4598-12-95

**Published:** 2013-08-26

**Authors:** Simon Welner, Nicole Hartwig Trier, Morten Frisch, Henning Locht, Paul Robert Hansen, Gunnar Houen

**Affiliations:** 1 Department of Clinical Biochemistry, Immunology and Genetics, Statens Serum Institut, Artillerivej 5, 2300, Copenhagen S, Denmark; 2 Present address: LEO Pharma A/S, Industriparken 55, 2750, Ballerup, Denmark; 3 Department of Drug Design and Pharmacology, Faculty of Health Sciences, University of Copenhagen, Universitetsparken 2, 2100, Copenhagen Ø, Denmark; 4 Department of Epidemiology Research, Statens Serum Institut, Artillerivej 5, 2300, Copenhagen S, Denmark; 5 Department of Rheumatology, Frederiksberg Hospital, 2000, Frederiksberg, Denmark

**Keywords:** Centromere protein-F, Autoantibodies, Immunofluorescence, ELISA, Cancer

## Abstract

**Background:**

Centromere protein-F (CENP-F) is a large nuclear protein of 367 kDa, which is involved in multiple mitosis-related events such as proper assembly of the kinetochores, stabilization of heterochromatin, chromosome alignment and mitotic checkpoint signaling. Several studies have shown a correlation between CENP-F and cancer, e.g. the expression of CENP-F has been described to be upregulated in cancer cells. Furthermore, several studies have described a significant correlation between the expression of autoantibodies to CENP-F and cancer.

**Methods:**

Autoantibodies to CENP-F were detected in a small number of samples during routine indirect immunofluorescence (IIF) analysis for anti-nuclear antibodies (ANA) using HEp-2 cells as substrate. Using overlapping synthetic peptides covering a predicted structural maintenance of chromosomes (SMC) domain, we developed an enzyme-linked immunosorbent assay (ELISA) for detection of CENP-F antibodies.

**Results:**

Analyzing the reactivity of the sera positive in IIF for CENP-F antibodies to overlapping CENP-F peptides, we showed that autoantibodies to several peptides correlate with the presence of antibodies to CENP-F and a diagnosis of cancer, as increased CENP-F antibody expression specific for malignant cancer patients to five peptides was found (A9, A12, A14, A16, A27). These antibodies to CENP-F in clinical samples submitted for ANA analysis were found to have a positive predictive value for cancer of 50%. Furthermore, the expression of cancer-correlated CENP-F antibodies seemed to increase as a function of time from diagnosis.

**Conclusion:**

These results conform to previous findings that approximately 50% of those patients clinically tested for ANA analyses who express CENP-F antibodies are diagnosed with cancer, confirming that these antibodies may function as circulating tumor markers. Thus, a peptide-based CENP-F ELISA focused on the SMC domain may aid in identifying individuals with a potential cancer.

## Background

Cancer occurs in many forms and the prognosis varies from good to extremely bad. For most forms of cancer, the prognosis is better, the earlier a diagnosis is established [1,2]. Consequently, screening for biomarkers is used to reveal early stages of disease or precancerous conditions. Such tumor markers can be substances released by cancer cells, circulating tumor cells or markers of an immune response against tumor components [2-6]. In general, immune responses against tumor cells are weak or non-detectable. However, in some cases characteristic autoantibodies are found in high titers in serum and have diagnostic value, e.g. paraneoplastic antibodies associated with paraneoplastic neurologic syndromes, which are believed to result from an autoimmune attack on neuronal tissue, spurred by similar neuronal antigens ectopically expressed in tumor cells [7,8].

Other examples of autoantibodies related to cancer include autoantibodies directed to centromere protein-F (CENP-F), also referred to as mitosin. These antibodies were originally suggested to be potential tumor markers by Casiano *et al*. [9]. They evaluated the clinical histories of 26 patients, who were found to express antibodies to CENP-F by indirect immunofluorescence (IIF). Of these 26 patients, 14 (54%) had various cancer types, while six (23%) had other disorders related to increased or abnormal cell proliferation, indicating a possible correlation between CENP-F antibodies and cancer. These findings were supported by Rattner *et al*. [10]. Analyzing CENP-F antibody expression in 36 sera from anti-CENP-F-positive patients, including the 26 patients originally examined by Casiano *et al*. [9], they reported that 22 of the 36 patients (61%) had neoplasms. Based on antibody screening, applying three larger fragments of the CENP-F protein, they concluded that the correlation between cancer diagnosis and CENP-F antibodies was more significant, the closer to the *C*-terminal end the autoantibodies showed reactivity. While the studies by Casiano *et al*. [9] and Rattner *et al*. [10] mainly described a correlation with breast and lung cancer, similar correlations have been described for other cancer types. For example, Bencimon *et al*. [11] detected a significant correlation between CENP-F antibody expression and non-Hodgkin's lymphoma (NHL) by screening 347 NHL patients along with 150 controls using a radioimmunoassay (RIA) and IIF. According to their findings, a significantly higher prevalence of CENP-F antibodies was detected in sera from NHL patients (7.2%) compared to controls (1.3%) by RIA (*P* < 0.01). In comparison, a prevalence of only 2.9% in sera from NHL patients compared with none of the sera from control patients was found when employing IIF, demonstrating a better sensitivity of the RIA technique. Similarly, a correlation between chronic graft versus host disease and the expression of antibodies to CENP-F has been described [12].

**Figure 1:**
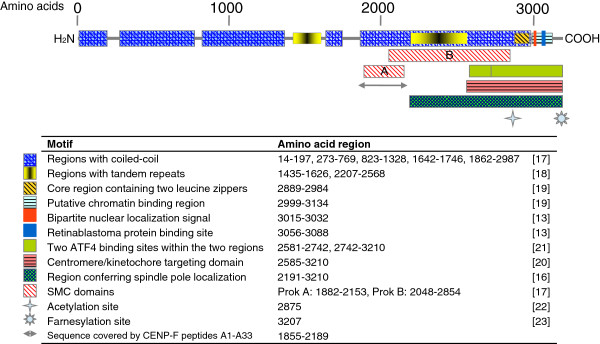
**Schematic presentation of CENP-F.** Domains, sequence motifs and the region studied in this article are indicated by amino acid number.

CENP-F is a 367 kDa protein of 3210 amino acids, which is involved in centromere formation and kinetochore organization during mitosis [13-16]. The protein is predicted to contain several structural features and motifs including coiled-coil, tandem repeats, leucine zippers and structural maintenance of chromosomes (SMC) domains [17-19]. Moreover, CENP-F has been experimentally shown to have several domains with distinct functions, including interaction with chromatin [19], retinoblastoma protein [13] and transcription factor ATF4 [20]. CENP-F contains a nuclear localization sequence [21], and it can be post-translationally modified by phosphorylation [13,21], acetylation [22] and farnesylation [23] (Figure 1).

Only limited information on the antigenic regions of CENP-F has been obtained. Studies by Rattner *et al*. [10] indicate that the *C*-terminal end is especially antigenic, however the exact regions remain to be determined. Recently, we characterized the reactivity of two independent monoclonal antibodies to CENP-F directed to regions of the predicted SMC prok A domain (amino acids 1882-2153), and showed that they recognize a linear epitope (NELSRIRSEKA, residues 1998-2008) in a putative coiled-coil region [24], confirming the antigenicity of this region.

In this study, we examined the reactivity of autoantibodies to CENP-F in patient sera to CENP-F peptides, found by routine IIF screening to exhibit the characteristic nuclear speckled-II (NSp-II) fluorescence pattern [25], which is characteristic for CENP-F antibodies. We focused on the predicted SMC prok A domain and designed overlapping 20-mer peptides, which were screened by ELISA for reaction with anti-CENP-F-positive patient sera. The results confirmed previous findings by Casiano *et al*. [9] and Rattner *et al*. [10], suggesting that approximately 50% of the NSp-II-positive sera had a cancer diagnosis. Moreover, antibody reactivity to specific CENP-F peptides could be correlated with this diagnosis.

## Results

### ANA results and diagnoses

**Figure 2:**
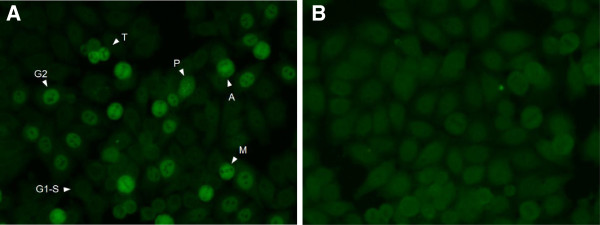
**Immunofluorescence on HEp-2 cells with anti-CENP-F-positive serum and control serum. (A)**. Reactivity of anti-CENP-F-positive patient serum (PS 26). Cells at different stages in the cell cycle are marked. G1-S: G1-S phase; G2: G2 phase; P: prophase; M: metaphase; A: anaphase; T: telophase. **(B)**. Reactivity of CENP-F-negative patient serum (Ctrl 5).

Out of 175,000 samples submitted for routine ANA screening, 42 revealed the characteristic NSp-II CENP-F pattern in IIF. These 42 samples were from 28 different individuals that were listed in groups according to their diagnoses (Table 1): invasive cancer (14 individuals, 50%), benign tumor (five individuals, 18%), and no registered neoplasias (nine individuals, 32%), mainly representing various connective tissue diseases. A representative NSp-II fluorescence pattern is illustrated in Figure 2, applying PS 26, while Ctrl 5 was used as negative control.

**Table 1:** Medical history and cancer cases of patients positive for NSp-II antibodies

**Group/**	**NSp-II signal in IIF**	**Time delay in months**	**Registered cancer cases**	**Additional medical history**
**sample ID**			**Topography/histology**	**Diagnosis**
*Invasive cancer*				
PS 1	Strong		Lung, small cell carcinoma	
PS 2.1	Strong	0	Cervix uteri, carcinoma in situ	Systemic lupus erythematosus
PS 2.2	Medium	26		
PS 2.3	Medium	74		
PS 2.4	Medium	97		
PS 4	Medium		Breast, Infiltrating duct carcinoma	
PS 6.1	Medium	0	Lung small cell carcinoma	Arthritis urica/Muscular rheumatism
PS 6.2	Strong	1		
PS 8.1	Medium	0	Lung, adenocarcinoma	Eye, benign tumor
PS 8.2	Medium	1		
PS 8.3	Medium	4		
PS 9.1	Medium	0	Ovary, clear cell adenocarcinoma	Peritoneum and lymph node metastasis
PS 9.2	Medium	1		
PS 9.3	Medium	10		
PS 9.4	Medium	18		
PS 12.1	Strong	0	Brain, unspecified tumor	Sjögren's syndrome/reumatoid arthritis
PS 12.2	Medium	25		
PS 12.3	Strong	51		
PS 12.4	Medium	53		
PS 13	Medium		Base of tongue, squamous cell carcinoma	Carrier of Human T-lymphotrophic virus-1/Reactive arthritis
PS 15	Strong		Esophagus, Squamous cell carcinoma	Rheumatoid arthritis
PS 16	Medium		Breast, infiltrating duct carcinoma	Bone or bone marrow, metastasis /Lymph node malignant tumor
PS 17.1	Medium	0	Breast, infiltrating duct carcinoma	Septicaemia
PS 17.2	Strong	14		
PS 17.3	Medium	59		
PS 18	Medium		Unknown site, adenocarcinoma	Lung, malignant tumor
PS 23	Strong		Base of tongue, squamous cell carcinoma	Oropharynx, malignant tumor
PS 27	Strong		Lymph node, diffuse large cell lymphoma	
*Benign tumor*				
PS 3	Weak			Liver transplantation/Face, benign tumor
PS 19	Weak			Skin, benign tumor/Sjögren's syndrome
PS 21	Medium			Breast, benign cystic tumor
PS 24	Strong			Neoplasia-associated polyneuropathy
PS 25	Weak			Breast, benign tumor
*No neoplasia*				
PS 5	Strong			Septicaemia
PS 7	Weak			
PS 10	Weak			
PS 11	Strong			Parkinson’s disease
PS 14	Medium			
PS 20	Medium			
PS 22	Weak			
PS 26	Strong			
PS 28	Medium			

### ELISA screening of anti-CENP-F-positive sera

**Figure 3:**
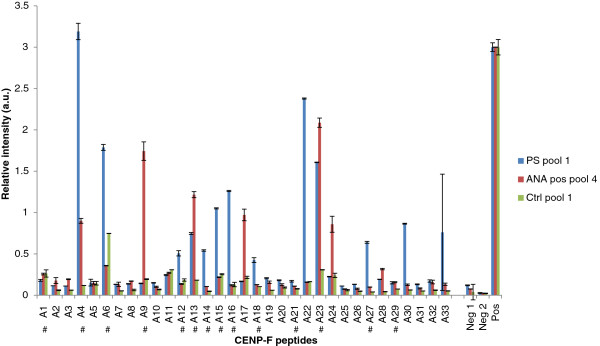
**Reactivity of anti-CENP-F-positive sera, ANA positive sera and control sera to overlapping CENP-F peptides analyzed by ELISA.** A. Reactivity of PS pool 1, ANA pos pool 4 and Ctrl pool 1 to CENP-F peptides spanning amino acid region 1855-2189. Blank wells without peptides (Neg 1) and serum pools (Neg 2) were applied for background determination. Peptide A22 vs. PS 18 was applied as positive control (Pos). # represents the peptides chosen for further analysis.

The reactivity of pools of anti-CENP-F-positive sera and pools of control sera to overlapping 20-mer peptides covering the CENP-F amino acid sequence 1855-2189 was analyzed by ELISA. Figure 3 illustrates the reactivity of PS pool 1 (represented by PS 1, PS 2.1, PS 9.2, PS 11, PS 15, PS 17.2, PS 18, PS 26), Ctrl pool 1 (represented by Ctrl 1-8) and ANA pos pool 4 (containing eight ANA-positive sera) to the 33 CENP-F peptides, while Additional file 1: Figure S1 illustrates the reactivity of all the pools screened. As seen, CENP-F antibody reactivity was found to multiple peptides. Antibody reactivity of PS pools was found to be focused around three regions A4-A6, A12-A16 and A21-A23. No distinct reactivity was found to these regions when analyzing reactivity of control pools (healthy donor pool and Ctrl pools), although all of the healthy donor pools showed weak reactivity to peptide A6. Similarly, three out of five ANA positive pools showed weak reactivity to peptides A9 and A17. Moreover, the region A22-A24 was found to be recognized by these pools as well. However, as these sera were CENP-F negative, these patterns are believed to be related to other nuclear proteins.

**Figure 4:**
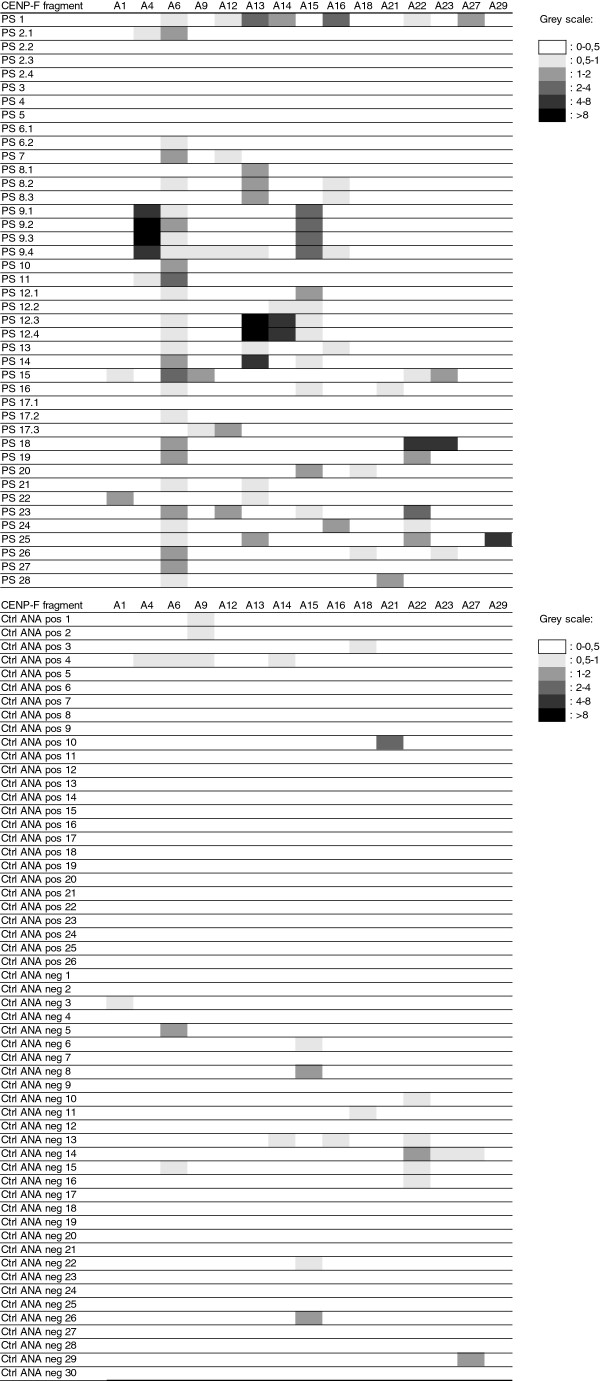
**Heat map of antibody profiles showing reactivity to selected peptides in individual patient serum samples.** Normalized reactivity is illustrated in grey scales. ANA positive samples 1-10 had a homogenous nuclear staining, 11-20 had a speckled nuclear staining and 21-26 had a centromere pattern).

Based on the collective findings, the peptides: A1, A4, A6, A9, A12-A16, A18, A21-A23, A27 and A29 were chosen for further analysis (marked with # in Figure 3). All of the available 42 samples from the 28 anti-CENP-F-positive patients and 86 Ctrl sera (56 from ANA screening (Figure 4), 20 from ELISA testing and 10 from healthy controls) were then tested individually for reactivity to the selected CENP-F peptides by ELISA. As seen in Figure 4, antibody reactivity was found to be concentrated around peptides A6, A13, A15 and A22, recognized by 23, 9, 7 and 7 anti-CENPF-positive patient sera, respectively. Of the 44 sera from 28 patients showing the NSp-II IIF pattern 7 did not react with any of the peptides. Three of these (PS3-5 were from patients, where only one sample was available, three (PS2.2-2.4) were from a patient, where only the first of four consecutive sera reacted in ELISA, and one (PS17.1) was the first of 3 consecutive sera, where the second (PS17.2) and third (PS17.3) showed reactivity with one and two peptides, respectively. The Ctrl sera (ANA-negative samples (n=30), ANA-positive samples (centromere (n=6), homogenous (n=10) and speckled (n=10) patterns) (Figure 4), CCP antibody-positive sera (n=10), DNA antibody-positive sera (n=10), healthy blood donors (n=10)) only occasionally showed weak reactivity to the CENP-F peptides (Figure 4 and results not shown). Most importantly, the sera showing the typical centromere pattern, which resembles the NSp-II pattern, were negative in ELISA.

### Correlation between diagnosis and antibody profile

In order to look for patterns of variation in the data set and in order to determine whether a correlation could be established between the CENP-F antibody reactivites and individual patient diagnoses, both a multivariate and a univariate analysis was used to evaluate the P-values of the contrasts between the diagnostic groups. For this, three scenarios were set: 1) all groups were regarded as different from each other; 2) the no neoplasia and the benign tumor groups were regarded as one; and 3) the benign tumor and the invasive cancer groups were regarded as one.

### Multivariate analysis of the fused matrix

**Figure 5:**
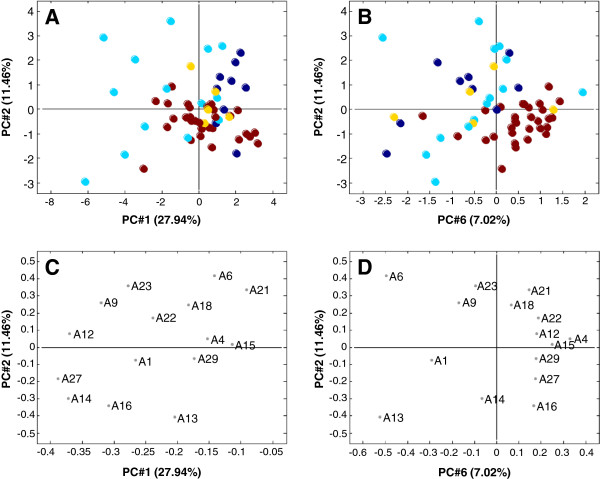
**Multivariate analysis of the fused matrix. (A)**: Score plot of PC#1 and PC#2. **(B)**: Score plot of PC#2 and PC#6. **(C)**: Loading plot of PC#1 and PC#2. **(D)**: Loading plot of PC#2 and PC#6. Percentage of complete variation of data described by the individual PCs is stated in parenthesis. Patient groups are illustrated as follows: brown: control, dark blue: no neoplasia, yellow: benign tumor, light blue: invasive cancer.

A principal component analysis (PCA), which describes the variation in the data set, was performed on the fused matrix and the scores of the resulting 15 PCs were analyzed in a spreadsheet in which the P-values for the contrasts between the diagnostic groups were calculated for each PC. Table 2 shows the P-values for the first six PCs. Out of all PCs only PC#1, PC#2, and PC#6 were found to describe statistically significant variations. The single significant contrast between the control and the benign tumor group in PC#4 was found to be the result of a single outlier and was therefore not taken into consideration. Figures 5A-D illustrate 2-dimensional projections of these three PCs and their respective loading plots. The diagnostic groups are represented by colors and can more or less be distinguished spatially from each other. Looking at the score plot in Figure 5A, the group with invasive cancer dominates the negative end of PC#1, while the control group forms a tilted ellipse around the center, intersected by a more vertical clustering of the no neoplasia group. The benign tumor group could be forming a cluster in the overlap zone of the other groups, but due to the limited number of samples in this group, a clear pattern cannot be established. Observed perpendicular to this in Figure 5B, the overall pattern is more diffuse except for the control group that has accumulated in the bottom right corner. Based on the results illustrated in Figure 5, the P-values displayed in Table 2 was calculated, from which the following statements are clear: PC#1 correlates significantly with the contrasts posed by the invasive cancer group, and to a lesser extent with the contrast between the control and the no neoplasia groups. PC#2 correlates exclusively and significantly with the contrasts posed by the control group, although only weakly when the tumor group is involved, and PC#6 correlates even stronger with the contrasts posed by the control group. The benign tumor group is not clearly distinguished from the other groups, partly because it is only represented by five samples, and partly because it assumes a very intermediate profile.

**Table 2:** PCA of the fused matrix

	**PC#1**		**PC#2**		**PC#3**		**PC#4**		**PC#5**		**PC#6**	
	**(27,94%)**		**(11,46%)**		**(9,29%)**		**(7,85%)**		**(7,53%)**		**(7,02%)**	
Control vs no neoplasia	**0,0139**	*	**0,0006**	***	0,1211		0,3422		0,0564		**0,0001**	***
Control vs benign tumor	0,6695		**0,0196**	*	0,0704		**0,0093**	**	0,8181		0,0146	
Control vs invasive cancer	**0,0014**	**	**0,0052**	**	0,1441		0,7088		0,1934		**0,0006**	***
No neoplasia vs benign tumor	**0,0114**	*	0,6004		0,0843		0,1410		0,5076		0,5799	
No neoplasia vs invasive cancer	**0,0005**	***	0,9463		0,9431		0,4794		0,5665		0,4471	
Benign tumor vs invasive cancer	0,0509		0,7425		0,1099		0,1423		0,6938		0,9850	
Control vs no neoplasia/benign tumor	**0,0300**	*	**0,0003**	***	0,7608		0,4215		0,1545		**0,0001**	***
No neoplasia vs invasive cancer	**0,0001**	***	0,8995		0,4505		0,7200		0,8283		0,5950	
Control vs benign tumor/invasive cancer	**0,0126**	*	**0,0032**	**	0,5683		0,1909		0,2926		**0,0003**	***
No neoplasia vs benign tumor/invasive cancer	**0,0018**	**	0,8412		0,5285		0,2759		0,4735		0,4262	

P-values for the contrasts between the four groups (Control, no neoplasia, begin tumor and invasive cancer) were calculated for each PC#1-#6 based on PCA of the fused matrix. *: P<0.05, **: P<0.01, ***: P<0.001.

Knowing what is described by the different PCs, the next step is to understand how the PCs themselves are described. To do this, the loading plots in Figure 5C and D were examined. The loading plots illustrate the projections of the 15 peptides onto the PCs in such a way that the greater the distance from zero a given peptide is placed along a PC, the greater “gravity” it imposes on the samples in the score plot. Therefore, the peptides in the negative end, of e.g. PC#1, are potentially responsible for pulling the samples towards this end, and since the negative end of PC#1 is dominated by invasive cancer samples, antibodies to these peptides are potentially correlated with invasive cancer. However, this cannot be unequivocally determined by an isolated view on the PCA, as a projection of a peptide can also pose a repulsive effect and thus inhabit a peripheral position in this capacity. To clarify this, the data obtained from the univariate analysis was considered.

### Univariate analysis of the fused matrix

P-values of the contrasts between the groups were calculated for each of the 15 peptides in the fused matrix using the Student’s t-test. The results are shown in Table 3. Beginning in the negative end of PC#1, antibodies to peptide A27 have the most negative loading score. At a first glance, it seems as if these antibodies pose a strong traction upon the invasive cancer group, but according to Table 2 they actually pose a strong repulsive action upon the no neoplasia group, while they do not distinguish between the other groups. In terms of antibody profile this means that the no neoplasia group has a significantly lower expression of antibodies against peptide A27 than the other groups. CENP-F antibodies to peptides A12 and A14 appear to have the same level of impact on PC#1. Their univariate profiles, however, are very different. Antibodies to peptide A12 do not specify much contrast, antibodies to peptide A14 pose a more hierarchical pattern, being most prevalent in the invasive cancer group, less so in the benign tumor and control groups and least in the no neoplasia group. The benign tumor group was not distinguished by antibodies to peptide A14. Closer to zero of PC#1 were CENP-F antibodies to peptide A9. According to Table 3 these antibodies are highly specific for the invasive cancer group, for which the expression was significantly elevated compared to the control and no neoplasia groups. Antibodies to peptide A16 were also more frequent in the invasive cancer group when compared to the no neoplasia group, but not when compared to the control group.

**Table 3:** Univariate analysis of the fused matrix

	**A1**	**A4**	**A6**	**A9**	**A12**	**A13**	**A14**	**A15**	**A16**	**A18**	**A21**	**A22**	**A23**	**A27**	**A29**
Control vs no neoplasia	0,60	0,08	0,00***	0,06	0,32	0,81	0,00**	0,87	0,00***	0,23	0,77	0,07	0,60	0,00**	0,00***
Control vs benign tumor	0,84	0,99	0,00***	0,30	0,12	0,36	0,06	0,09	0,51	0,32	0,28	0,1	0,55	0,09	0,18
Control vs invasive cancer	0,04*	0,63	0,00***	0,00***	0,05	0,05*	0,01*	0,18	0,49	0,00**	0,00**	0,29	0,01**	0,44	0,92
No neoplasia vs benign tumor	0,88	0,36	0,76	0,19	0,68	0,61	0,29	0,27	0,19	0,29	0,57	0,05*	0,91	0,03*	0,13
No neoplasia vs invasive cancer	0,16	0,23	0,83	0,01**	0,08	0,27	0,01**	0,34	0,02*	0,56	0,11	0,07	0,06	0,00**	0,01*
Benign tumor vs invasive cancer	0,26	0,83	0,55	0,35	0,05*	0,72	0,07	0,06	0,53	0,00**	0,42	0,60	0,11	0,09	0,36
Control vs no neoplasia/benign tumor	0,62	0,21	0,00***	0,55	0,13	0,82	0,00***	0,34	0,01**	0,58	0,84	0,70	0,48	0,00***	0,47
No neoplasia vs invasive cancer	0,11	0,31	0,66	0,02*	0,02*	0,32	0,00**	0,09	0,04	0,16	0,13	0,33	0,02*	0,00**	0,65
Control vs benign tumor/invasive cancer	0,11	0,69	0,00***	0,00**	0,30	0,06	0,11	0,63	0,77	0,05*	0,00**	0,15	0,04*	1,00	0,56
No neoplasia vs benign tumor/invasive cancer	0,23	0,22	0,97	0,02*	0,20	0,28	0,03*	0,67	0,03*	0,99	0,12	0,04*	0,14	0,1*	0,07

P-values of the contrasts between the groups were calculated for each of the 15 peptides in the fused matrix. Italics correlate negatively. *: P<0.05, **: P<0.01, ***: P<0.001.

Looking at PC#2 and PC#6, especially antibodies to peptides A6 and A13 were placed in the periphery. According to the univariate data analysis, antibodies to peptide A6 were interesting, as they posed significant contrasts for all pairs of groups in which the control group was included, meaning that the expression of antibodies to A6 was significantly raised in all anti-CENP-F-positive sera compared to the controls.

### Multivariate analysis of the complete matrix

A PCA of the complete matrix was performed to investigate the change in antibody profile as a function of the time of blood sampling. This was performed by analyzing the six patients that were represented by more than one blood sample (PS 2, PS 6, PS 8, PS 9, PS 12 and PS 17). Additional file 2: Figure S2 illustrates the 2D score plots of PC#1 and PC#2 for each of the six patients and reveals a general pattern of migration towards the negative end of PC#1 as a function of blood sampling time, suggesting a correlation between diagnosis/tumor stage and sample time.

## Discussion

In this study, we analyzed the correlation between diagnoses and the presence of antibodies to CENP-F, determined by IIF using HEp-2 cells. We confirmed that 50% of anti-CENP-F-positive individuals had a cancer diagnosis, as reported by others [9-11,25,26]. The remaining 50% mainly had a benign tumor or a connective tissue disease.

Moreover, we analyzed the presence of CENP-F antibodies directed to the predicted SMC A domain using overlapping 20-mer peptides. In this regard, it must be emphasized that we most likely do not detect antibodies to conformational and discontinuous epitopes and that we only used peptides from the SMC A domain. This is a major difference compared to earlier studies using fragments of CENP-F. Moreover, the study material was small, which is a limitation to the conclusions drawn here. Despite these limitations, the results were significant and may have important implications for future studies.

The ELISA results revealed that CENP-F antibody expression to eight of the 15 screened peptides (A1, A6, A9, A13, A14, A18, A21 and A23) was increased significantly in the invasive cancer compared to the control group. Moreover, CENP-F antibody expression to five peptides (A9, A14, A16, A27 and A29) was increased significantly in the invasive cancer group compared to the no neoplasia group. Especially the expression of CENP-F antibodies to peptides A9 and A14 was elevated in the invasive cancer group exclusively. CENP-F antibody reactivity to other peptides, such as A18, A21 and A23, also correlated with invasive cancer diagnoses, but not in an exclusive manner. CENP-F antibody expression to peptide A6 exhibited a strong correlation with all of the anti-CENP-F-positive groups (invasive cancer, benign tumor and no neoplasia) compared to the controls. Similarly, CENP-F antibody expression to peptide A27 was increased in all groups except from the no neoplasia group, a characteristic that was partly shared by peptide A14 and A16 as well.

The peptides A4 and A15 did not represent any contrast at all, but have still been included in the analysis, as they did display reactivity during the preliminary pool screenings. This indicated that their antibody specificity was not correlated with any of the groups. For peptide A15, an epitope has been finely mapped in a parallel study in which two monoclonal antibodies recognized this peptide out of the 33 peptides (A1-A33) [24].

More generally, as seen in Table 3, it could be interpreted that the CENP-F antibody expression profile was generally increased in the invasive cancer group when compared to both the control and the no neoplasia groups. The benign tumor group was only vaguely distinguishable from the other groups, an interpretation that did not contain much value due to the limited number of patients in that group.

The correlation between CENP-F antibodies and cancer is an interesting phenomenon. Also, several correlations between other proteins of the CENP family and autoimmune illnesses have been reported. Mahler *et al.*[27] described a study of systemic sclerosis patients, who developed an antibody response against histone H3 that also induced reactivity against CENP-A and CENP-B through intra- and intermolecular epitope spreading. Other patients with systemic sclerosis have been described to express autoantibodies against CENP-E, CENP-I and CENP-O [28-30]. Hsu *et al.*[31] described the presence of CENP-H antibodies in patients with Sjögren's syndrome and Ford *et al*. [32] described the presence of CENP-D IgM autoantibodies in a patient prior to the development of CREST (calcinosis, Raynaud's phenomenon, esophageal dysmotility, sclerodactyly, telangiectasias) symptoms.

The factors eliciting an immune response against CENP-F are unknown. Cancer is a disease caused by excessive cell proliferation and metastasis [1,6,33]. CENP-F is involved in cell division and proliferation [13-15,17,20,34-39] and it has been described that the expression of CENP-F is markedly elevated in some cancers [40-46]. In a study of nasopharyngeal carcinoma patients this over-expression was found to be localized preferentially in the cancer cells of the invasive front, indicating a potential role in promoting tumor invasion [40]. It is likely that cells of such a rampant cell cluster would eventually lyse and release CENP-F, thus making it available for the immune system. Studies of ANA in systemic autoimmune diseases have suggested that the autoimmune response is indeed antigen-driven [47] and ANAs have previously been described in several types of cancer including ovarian and lung cancer [48-51].

In conclusion, we have developed an ELISA system for detecting CENP-F autoantibodies and we confirm the conclusion reached by Fritzler *et al*., that although such antibodies have a low sensitivity, the positive predictive value for cancer in clinical samples submitted for ANA analyses is approximately 50% and the positive predictive value for neoplasia even higher. In future studies, we would like to extend these studies to the whole CENPF sequence using longer peptides and to larger patient cohorts.

## Methods

### Peptide fragments for analysis

The human CENP-F sequence (Uniprot KB ID: P49454), comprising 3210 amino acids, was used to construct 33 overlapping peptides, A1-A33, which span the amino acid sequence 1855-2189 (Figure 1). The length of each peptide was 20 amino acids, with an overlap of 10 amino acids to the next peptide. At the *N*-terminus, a cysteine residue was added in order to have the possibility to screen the peptides using CovaLink™ technology (see peptide ELISA). The peptides A1-A16, A18, A20-A33 were purchased from Schäfer-N (Copenhagen, Denmark), while peptides A17 and A19 were synthesized as described elsewhere [24]. The peptides, A1-A33, are listed in the supplementary table (Additional file 3: Table S1).

### Serum samples

Sera were routinely screened for the presence of antinuclear antibodies (ANA) by IIF using HEp-2 cells as described below. Out of approximately 175,000 samples, submitted for routine ANA screening over a period of approximately 8 years, 42 samples from 28 patients were positive for the NSp-II pattern, characteristic for CENP-F antibodies [25]. Most patients were represented by a single serum sample, while a few were represented by up to four samples (Table 1). Each individual anti-CENP-F-positive sample was given a unique identification code, PS, followed by a number from 1-28 referring to the patient. A decimal number from 1-4 was added to the samples from patients represented more than once, 1 referring to the oldest sample and 4 to the most recent sample. The following six patients were represented more than once: PS 2 (PS 2.1-2.4), PS 6 (PS 6.1-6.2), PS 8 (PS 8.1-8.3), PS 9 (PS 9.1-9.4), PS 12 (PS 12.1-12.4) and PS 17 (PS 17.1-17.3). In addition, 30 samples that all tested negative for ANA were used as controls. These are referred to as Ctrl 1-30. A list of the medical histories and registered cancer diagnoses among patients positive of CENP-F antibodies was acquired retrospectively from the National Board of Health (Table 1). Based on this, analyzed samples were grouped as follows: Controls (30/58), patients without neoplasia (9/58), patients with benign tumors (5/58), patients with invasive cancer (14/58). In addition 10 sera positive for CCP antibodies, 10 sera positive for DNA antibodies and 10 sera from healthy blood donors were analysed by ELISA. All serum samples were acquired from the serum bank at Statens Serum Institut.

### Pretreatment of serum for inactivation of the complement system

Non-specific binding in serum screening experiments is a well-known cause of measurement uncertainty and false positive results. Aluminum hydroxide pre-treatment has been found to reduce non-specific binding in ELISA (Güven *et al.*, unpublished data). Pretreatment of sera was performed by mixing 1:1 with aluminum hydroxide (Alhydrogel 2%, Brenntag Biosector, Frederikssund, Denmark). After having incubated on a shaker for 1 h at room temperature (RT) the sample was centrifuged, the supernatant diluted 1:100 in Tris-Tween-NaCl (TTN) buffer (0.05 M Tris, 1% Tween 20, 0.3 M NaCl, pH 7.5) and applied for ELISA.

### Indirect immunofluorescence for anti-nuclear antibodies

Sera were diluted in phosphate-buffered saline (PBS) (10 mM Na_2_HPO_4_/NaH_2_PO_4_, 0.15 M NaCl, pH 7.2) (1:160) and 20 μl were applied to wells of glass slides with fixed HEp-2 cells (Immunoconcepts, Sacramento, CA, USA) and incubated at RT in a humidified chamber. Wells were rinsed with PBS, and 20 μl FITC-conjugated rabbit Igs against human IgG (DAKO, Copenhagen, Denmark), diluted in PBS (1:40), were applied to the wells and incubated 30 min. The wells were rinsed with PBS and the slides were mounted with coverslips and inspected with a fluorescence microscope (Aristoplan (Leica), BH2 (Olympus) or Eurostar (Euroimmun)). The immunofluorescence pattern and the intensity were graded by intensity of 0 (negative), +1 (weak), +2 (medium), 3+ (strong) and recorded essentially as described elsewhere [52].

### Peptide enzyme-linked immunosorbent assay (ELISA)

In initial experiments, CovaLink plate immobilization was compared with Maxisorp plate adsorption. The covalink plates showed too high background and further experiments were done with simple adsorption in Maxisorp plates. The wells of a 96-well Maxisorp microtitre plate (Nunc, Roskilde, Denmark) were coated with 1.5 μg/well peptide dissolved in carbonate buffer (15 mM Na_2_CO_3_, 35 mM NaHCO_3_, 0.001% phenol-red, pH 9.6) and incubated over night at 4°C. Wells were rinsed with TTN buffer and incubated with sera (with or without aluminum hydroxide pretreatment) diluted in TTN (1:200) for 1 h. The wells were rinsed with TTN (3 × 5 min) and incubated 1 h with alkaline phosphatase-conjugated goat IgG against human IgG (Sigma Aldrich, Steinheim, Germany) followed by three washes with TTN buffer (5 min each). Bound antibodies were quantified using *para*-nitrophenylphosphate (1 mg/mL) (Sigma Aldrich) dissolved in alkaline phosphatase substrate buffer (1 M diethanolamine, 0.5 mM MgCl_2_, pH 9.8). After sufficient color reaction, the absorbance was measured at 405 nm on a Thermomax microtitre plate reader (Molecular Devices, Menlo Park, CA, USA). All samples were analyzed in duplicate.

### ELISA for CCP and DNA antibodies

CCP antibodies were determined with the CCP2 ELISA kit (Eurodiagnostica, Malmö, Sweden) and DNA antibodies were determined by ELISA with the VarElisa kit (Thermo Scientific, Uppsala, Sweden) following the instructions of the manufacturers.

### Normalization of data

All sera-pool screenings were normalized relative to internal reference sera and to each other.

### Multivariate data analysis

Multivariate data analysis was performed using the principal component analysis (PCA) software, LatentiX (Latent5, downloaded from latentix.com). Prior to this, the normalized data for the individual screenings was mounted in two different data matrices. One matrix, referred to as *the complete matrix*, contained data from all the individual screenings, while the other, referred to as *the fused matrix*, contained the data from the patients represented by multiple samples fused into one average data unit per patient. This was done to allow equal impact of each patient. Both matrices were transformed logarithmically prior to analysis in order to approximate a Gaussian distribution. In LatentiX, the data was transformed using the autoscale function and the PCA models were calculated without validation.

## Abbreviations

ANA: Antinuclear antibodies; CENP-F: Centromere protein-F; ELISA: Enzyme-linked immunosorbent assay; IIF: Indirect immunofluorescence; NHL: Non-Hodgkin's lymphoma; NSp-II: Nuclear speckled pattern-II; PBS: Phosphate-buffered saline; PC: Principal component; PCA: Principal component analysis; RIA: Radioimmunoassay; RT: Room temperature; SMC: Structural maintenance of chromosomes; TTN: Tris-Tween-NaCl.

## Competing interests

The authors declare no competing interests.

## Authors’ contributions

SW conducted experiments as well as carried out data analysis and manuscript writing. NHT conducted preliminary experiments, peptide synthesis, participated in intellectual discussion of the data and manuscript writing. PRH and GH conducted design study, participated in intellectual discussion of the data and manuscript writing. MF and HL collected patient sera and retrieved medical patient histories. All authors read and approved the final manuscript.

## Supplementary Material

Additional file 1: Figure S1 Heat map of antibody profiles showing reactivity of sera pools to peptides 1-33. Sera pools PS pool 1, PS pool 2, individual anti-CENP-F-positive pools PS 12.1, P24 and P27 (19 in total), Ctrl pool 1, ANA negative pools 1-5, healthy donor pools 1-4 and ANA positive pools 1-5 were analyzed for reactivity. Normalized reactivity is illustrated in grey scales. Click here for file

Additional file 2: Figure S2 Multivariate analysis of the complete matrix. Percentage of complete variation of data described by the individual PCs is stated in parenthesis. Consecutive blood samples for patients with multiple samples are highlighted in yellow. A: PS 17. B: PS 12. C: PS 9. D: PS 8. E: PS 2. F: PS 6. Patient groups are illustrated as follows: brown: control, dark blue: no neoplasia, yellow: benign tumor, light blue: invasive cancer. Click here for file

Additional file 3: Table S1 Overlapping CENP-F peptides applied for screening. Click here for file
