# Supplementary figures and images for: Correlation between centromere protein-F autoantibodies and cancer analyzed by enzyme-linked immunosorbent assay
Source: Mol Cancer. 2013 Aug 26;12:95. doi: 10.1186/1476-4598-12-95 (PMC3844405; doi:10.1186/1476-4598-12-95)

## Slide 1
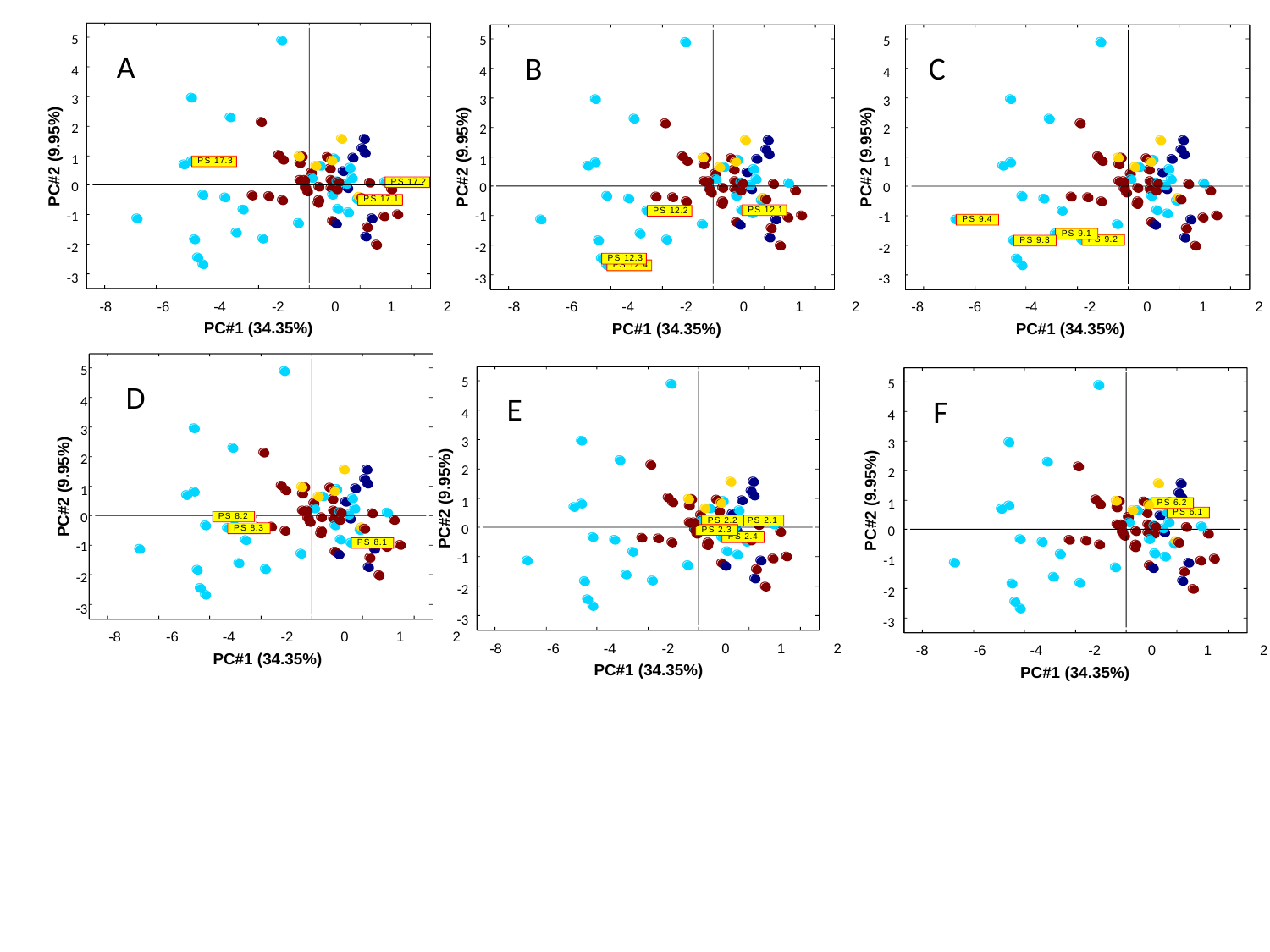

5
A
4
3
2
PC#2 (9.95%)
1
0
-1
-2
-3
	-8	-6	-4	-2	0	1	2
PC#1 (34.35%)
5
B
4
3
2
PC#2 (9.95%)
1
0
-1
-2
-3
	-8	-6	-4	-2	0	1	2
PC#1 (34.35%)
5
C
4
3
2
PC#2 (9.95%)
1
0
-1
-2
-3
	-8	-6	-4	-2	0	1	2
PC#1 (34.35%)
5
D
4
3
2
PC#2 (9.95%)
1
0
-1
-2
-3
	-8	-6	-4	-2	0	1	2
PC#1 (34.35%)
5
E
4
3
2
PC#2 (9.95%)
1
0
-1
-2
-3
	-8	-6	-4	-2	0	1	2
PC#1 (34.35%)
5
F
4
3
2
PC#2 (9.95%)
1
0
-1
-2
-3
	-8	-6	-4	-2	0	1	2
PC#1 (34.35%)

Supplement: Additional file 2: Figure S2 — Multivariate analysis of the complete matrix. Percentage of complete variation of data described by the individual PCs is stated in parenthesis. Consecutive blood samples for patients with multiple samples are highlighted in yellow. A: PS 17. B: PS 12. C: PS 9. D: PS 8. E: PS 2. F: PS 6. Patient groups are illustrated as follows: brown: control, dark blue: no neoplasia, yellow: benign tumor, light blue: invasive cancer. [file 1476-4598-12-95-S2.pptx]
