# Supplementary material for: Correlation between centromere protein-F autoantibodies and cancer analyzed by enzyme-linked immunosorbent assay
Source: Mol Cancer. 2013 Aug 26;12:95. doi: 10.1186/1476-4598-12-95 (PMC3844405; doi:10.1186/1476-4598-12-95)
Supplement: Additional file 3: Table S1 — Overlapping CENP-F peptides applied for screening. [file 1476-4598-12-95-S3.docx]

| **Peptide** | **Amino acid sequence** |
| --- | --- |
| A1 | CPMDFLGNQEDIHNLQLRVKE |
| A2 | CIHNLQLRVKETSNENLRLLH |
| A3 | CTSNENLRLLHVIEDRDRKVE |
| A4 | CVIEDRDRKVESLLNEMKELD |
| A5 | CSLLNEMKELDSKLHLQEVQL |
| A6 | CSKLHLQEVQLMTKIEACIEL |
| A7 | CMTKIEACIELEKIVGELKKE |
| A8 | CEKIVGELKKENSDLSEKLEY |
| A9 | CNSDLSEKLEYFSCDHQELLQ |
| A10 | CFSCDHQELLQRVETSEGLNS |
| A11 | CRVETSEGLNSDLEMHADKSS |
| A12 | CDLEMHADKSSREDIGDNVAK |
| A13 | CREDIGDNVAKVNDSWKERFL |
| A14 | CVNDSWKERFLDVENELSRIR |
| A15 | CDVENELSRIRSEKASIEHEA |
| A16 | CSEKASIEHEALYLEADLEVV |
| A17 | CLYLEADLEVVQTEKLCLEKD |
| A18 | CQTEKLCLEKDNENKQKVIVC |
| A19 | CNENKQKVIVCLEEELSVVTS |
| A20 | CLEEELSVVTSERNQLRGELD |
| A21 | CERNQLRGELDTMSKKTTALD |
| A22 | CTMSKKTTALDQLSEKMKEKT |
| A23 | CQLSEKMKEKTQELESHQSEC |
| A24 | CQELESHQSECLHCIQVAEAE |
| A25 | CLHCIQVAEAEVKEKTELLQT |
| A26 | CVKEKTELLQTLSSDVSELLK |
| A27 | CLSSDVSELLKDKTHLQEKLQ |
| A28 | CDKTHLQEKLQSLEKDSQALS |
| A29 | CSLEKDSQALSLTKCELENQI |
| A30 | CLTKCELENQIAQLNKEKELL |
| A31 | CAQLNKEKELLVKESESLQAR |
| A32 | CVKESESLQARLSESDYEKLN |
| A33 | CLSESDYEKLNVSKAL |

**Table S1** Overlapping CENP-F peptides applied for screening.
